# Long-Term Experimental Evaluation of In Situ Laser-Fenestrated Versus Custom-Made Fenestrated Endografts

**DOI:** 10.1177/15266028251350485

**Published:** 2025-07-05

**Authors:** Florian Elger, Marcello Silvano, Teodora Krasimirova Ormandzhieva, Ingo Kutschka, Giovanni Battista Torsello, Giovanni Federico Torsello

**Affiliations:** 1 Department of Cardiac, Thoracic and Vascular Surgery, University Medical Center Göttingen, Göttingen, Germany; 2 Department of Radiology, University Medical Center Göttingen, Göttingen, Germany

**Keywords:** complex aortic aneurysm, In situ laser fenestration, custom-made endograft, fatigue test

## Abstract

**Objective:**

This study evaluates the biomechanical performance and long-term durability of in situ laser fenestration (ISLF) compared to custom-made fenestrated endografts (CMD) using a controlled bench-top experimental setup.

**Methods:**

In situ, laser fenestration was performed in a RelayPro Dacron endograft using a 2.0 mm Turbo Elite OTW Laser Atherectomy catheter. Ten BeGraft Bridging Stent-Grafts (BSG) were implanted in 5 ISLF and 5 CMD Zenith fenestrations and subjected to 50 million fatigue cycles. Fenestrations were evaluated radiographically and microscopically for shape, size, fraying, endograft tearing, and BSG integrity.

**Results:**

Fenestration areas (median 8.1 mm^2^) and axis lengths (median long axis: 4.03 mm, median short axis: 2.33 mm) in the ISLF group were significantly less than CMD fenestrations (median area: 24 mm^2^; median long axis: 5.83 mm, median short axis: 5.8 mm) immediately post-manufacturing (p=0.002), but increased significantly after stenting and fatigue (p=0.002). Tearing was observed in 3 of 5 ISLF after ballooning but resolved after BSG implantation. BSG integrity was superior in the ISLF group, with no all-layer defects, while 3 of the 5 tested BeGrafts used in the CMD group revealed fabric defects with partial fracture of the stent in 2 (p=0.007).

**Conclusion:**

ISLF fenestrations demonstrated comparable durability to CMD fenestrations after long-term fatigue testing, with distinct advantages in BSG integrity preservation. These findings support ISLF as a viable option for urgent treatment, although further research is warranted to validate these results.

**Clinical Impact:**

In-situ laser fenestration is a feasible technique for urgent complex aortic repair when customized or off-the-shelf devices are unavailable or technically unfeasible. However, long-term mechanical stability and durability under fatigue testing have not been previously evaluated. This study is the first to directly compare the long-term mechanical performance of industry-made custom fenestrations with in-situ laser fenestrations using a fatigue model. Results demonstrate non-inferiority of in-situ laser fenestrations, supporting their use as an effective off-label strategy for emergency or bailout interventions.

## Introduction

Complex abdominal aortic aneurysms (AAAs) pose a significant challenge in vascular surgery due to their anatomical complexities and the risk of rupture if left untreated. Endovascular aneurysm repair (EVAR) has emerged as a less invasive alternative to open surgery, offering reduced perioperative morbidity and shorter recovery times.^1^ For complex AAAs, fenestrated endovascular aortic repair (FEVAR) is the preferred approach, as it allows for the preservation of blood flow to aortic branch vessels while effectively excluding the aneurysm sac from systemic circulation.^2^

FEVAR involves the use of fenestrated endografts, which are custom-manufactured to match the patient’s unique vascular anatomy. However, the customization process necessitates a production time of several weeks, rendering FEVAR unsuitable for patients requiring urgent treatment.^3^ In such cases, alternative strategies have been proposed, including the use of standard endografts modified in situ to accommodate branch vessels. Among these techniques, in situ laser fenestration (ISLF) has gained traction due to its adaptability and potential to provide a rapid solution in emergency settings.^4^

Despite its growing use, the long-term performance of in situ laser fenestration remains underexplored, particularly when compared to standard custom-made devices (CMD). Understanding the biomechanical and dynamic outcomes of these approaches is critical for optimizing FEVAR late results and guiding clinical decision-making. This study aims to evaluate the long-term performance of in situ laser fenestration by comparing its biomechanical and dynamic outcomes with those of the standard CMD in a controlled bench-top experimental setup.

## Methods

### In Situ Laser Fenestration

Five fenestrations are performed with an Excimer Laser System, using a 2.0 mm Turbo Elite OTW Laser Atherectomy catheter (Spectranetics, Colorado Springs, CO, USA) at a fluency of 45 mJ/mm^2^ and pulse rate of 25 Hz. These settings are based on our clinical protocol for treating patients with in situ fenestration in an emergency. Fenestrations are then pre-dilatated with a 6 × 20 mm Sterling balloon catheter (Boston Scientific, Marlborough, USA) inflated up to 10 Atm and kept inflated for 20 seconds before the bridging stent graft(BSG) is implanted.

### Implantation of Bridging Stent

Totally, ten 6 × 58 mm balloon-expandable BeGraft Peripheral stent grafts (Bentley InnoMed GmbH, Hechingen, Germany) are deployed in accordance with clinical practice. Five are implanted in a polyester sheet (Cook Medical, Bloomington, IN, USA) with 6 mm diameter fenestrations and 5 in a RelayPro (Terumo Aortic, Sunrise, FL, USA) sheet after in situ Laser fenestration performed as described above. After release, the stent grafts are flared with a 10 × 20 mm Admiral Xtreme PTA catheter (Medtronic, Minneapolis, USA) for 30 seconds. All BSGs are implanted at a room temperature of 20°C to 22°C and in a water bath with a water temperature of 37°C, which are standard conditions to ensure comparability with former in vitro tests^5^ and in vivo scenarios encountered in our clinical practice.

### Fatigue Test

**Figure 1.**
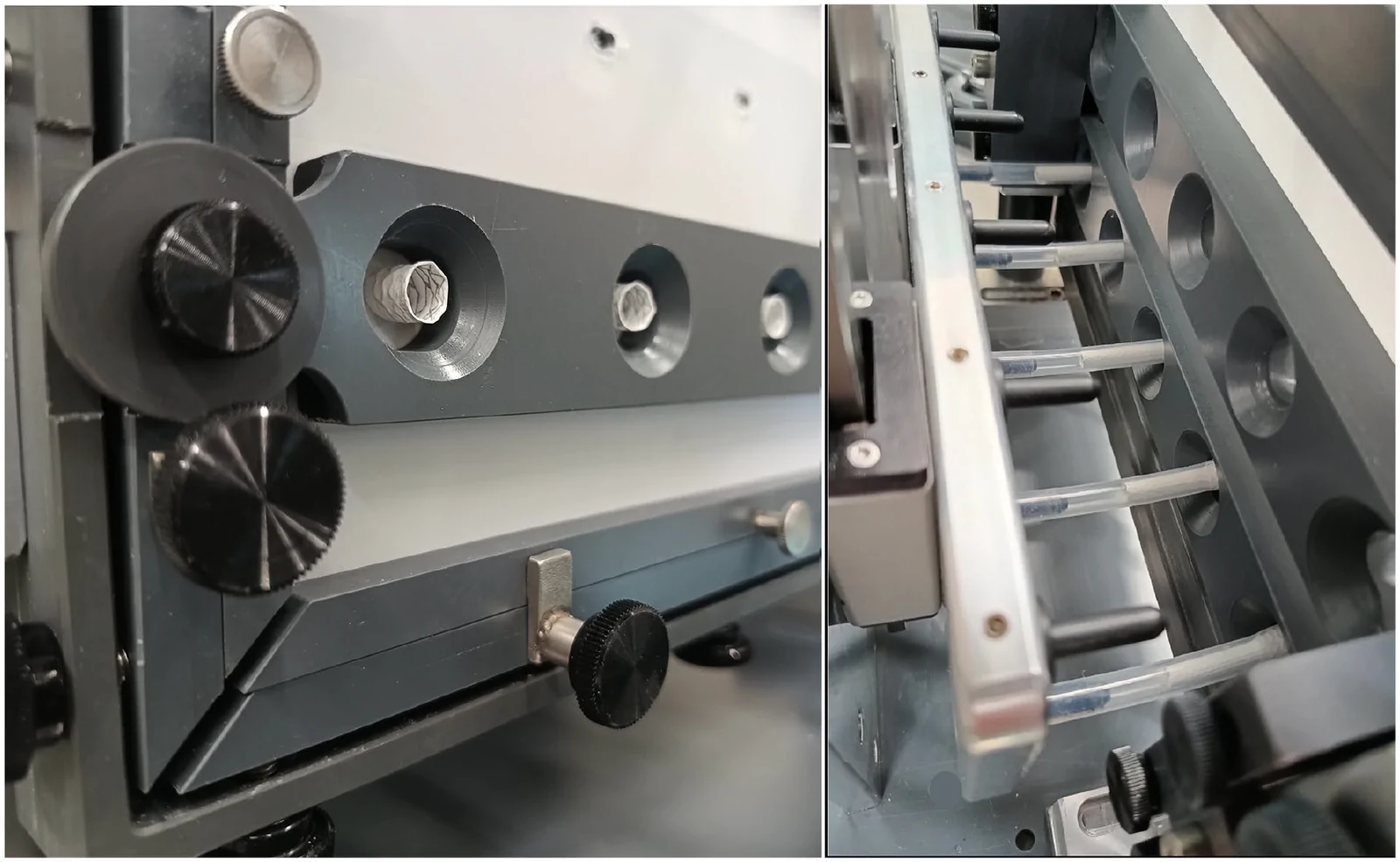
Test setup with tensioned RelayPro fabric housed in a frame. Brackets support the fabric on both sides of the fenestration. Begraft stents are deployed within silicone tubes simulating the target vessels and are flared on the aortic side of the fenestration.

The fenestrated sheet with the implanted BSGs is then mounted in a frame (Figure 1). As described by other authors,^5^ we created a setting simulating the in vivo situation of respiratory cyclic movement with 15 cycles/min. This setup reflects the physiological cranio-caudal motion of the kidneys during respiration, which can lead to stent fatigue, fracture, or dislocation over time. The peripheral ends of the stents are placed in silicone tubes simulating target vessels such as renal arteries. The BSGs are then connected with a testing machine and subjected to oscillating craniocaudal angulations of ±15 mm. The BSG primary stress point is at the fenestration area, while the distal portion is mechanically not affected. A controller is used to set the speed and to count the performed cycles. The frequency of the movement was 3.5 Hz.

### Radiographic and Microscopic Evaluation

Resistance to fatigue is evaluated by digital X-ray and microscopy. All images are independently evaluated by 2 investigators.

Digital radiographic imaging is performed using a Ysio X.pree X-ray system (Siemens Healthineers, Erlangen, Germany). Applied X-ray parameters were: 50 KV, 0.2 seconds and 1 mAs.

Radiologic imaging is performed before the stress test and then at 1, 5, 25, 40, and 50 million cycles. To assess stent expansion, the diameter at the level of the fenestration is compared with the diameter of the expanded stent outside the flaring region.

The following findings are investigated: >30% stent stenosis and stent frame fracture. If any are observed, maximum cycles free from fracture and freedom from stent stenosis are evaluated over time.

Microscopic imaging is performed before implantation of the BSG and during the fatigue test 1, 5, 25, 40, and 50 million cycles in both groups. 50 million cycles simulate a period of approximately 6.8 years at an estimated breathing rate of 14 to 15/min

A microscope camera with a ×140 magnification and measuring software (Dinolite AM8917MZTL and **DinoCapture 2.0**, Dunwell Tech. Inc., Torrance, USA) is used for image recording using a calibration slide (AmScope, USA). For area measurements, we used the ImageJ 1.54h software (public domain) after placing an ROI around each fenestration. Images are acquired from the aortic and the peripheral side of the sheet, with a focus on stent fabric, metal frames, and the interface between the polyester sheet and covered stent. The following morphological aspects are investigated:

Fenestration shape (round, elliptical, square, or slit-like) before and after fatigue

Long and short axis of the fenestration

Fenestration area before and after fatigue

Fraying defined as increased number (>5) of damaged fabric fibers visible before and after fatigue

Fabric tearing before and after fatigue

PTFE changes (any alteration of the PTFE coating)

PTFE defect (breakpoint) with exposure of the stent metal

During the fatigue test the following parameters are assessed: maximum number of cycles without coating alterations that is, freedom from initial PTFE changes (FIC), and freedom from PTFE breakpoint (FBP). The cycle number of the last imaging showing no alteration of the PTFE fabric is used for determining the freedom from FIC and FBP.

Endpoint of the evaluation is the long-term device failure, defined as a composite of stent fracture, stent stenosis, PTFE all-layer defect, and tearing after fatigue.

### Statistical Analysis

Statistical data analysis was performed with the software SPSS Statistics for Windows (IBM release 24, Chicago, IL, USA). Categorical variables are expressed as frequency and percentage, whereas continuous variables are presented with medians, minimum and maximum, as well as 25 and 75 percentiles. Because of the small sample sizes, non-normal distributions were assumed. The Pearson Chi-squared test and Mann-Whitney U test were employed for categorical and continuous variables, respectively. Kaplan-Meyer estimates and log-rank test were employed to evaluate the freedom from breakpoint and the freedom from PTFE changes. Differences are considered significant at p<0.05.

## Results

Fenestration shape was round in all cases of the CMD group before and after fatigue testing. Before fatigue testing, 4 fenestrations of the ISLF group had a square and 1 an elliptical shape. After fatigue testing, all CMD fenestrations had elliptical shapes. Size measurements as well as microscopic and radiographic evaluation of the fenestration of both groups are summarized in Table 1.

**Table 1.** Summarized Results of the Fatigue Tests.

Fenestrations before fatigue test			
	ISLF	CMD	*P*
Fenestration features N (%)			
Tearing	3 (60)	–	.038
Fraying	4 (80)	–	.010
Shape			
Circular	–	5 (100)	
Elliptical	1 (20)	–	.007
Squared	4 (80)	–	
Fenestration measures Me (IQR)			
Long axis (mm)	4.03 (4.49–3.48)	5.83 (5.91–5.72)	.002
Short axis (mm)	2.33 (2.48–2.18)	5.80(5.88–5.69)	.002
Area (mm^2^)	8.10 (8,55–5.85)	24,0 (24.42–23.78)	.002
Fenestrations and stent outcomes after fatigue test			
	ISLF	CMD	*P*
Fenestration features N (%)			
Tearing	–	–	–
Fraying	5 (100)	–	.002
Shape			
Circular	–	5 (100)	
Elliptical	5 (100)	–	.002
Squared	–	–	
Fenestration measures Me (IQR)			
Long axis (mm)	6.09 (7.14–5.70)	7.03 (7.18–6.95)	.527
Long axis increase after fatigue (%)	34.5 (45.0–30.8)	16.5 (19.5–16.2)	.002
Short axis (mm)	3.40 (3.99–3.12)	6.91 (7.12–6.89)	.002
Short axis increase after fatigue (%)	35.1 (40.9–24.5)	16.8 (18.9–15.6)	.002
Area (mm^2^)	17.50 (21.38–16.25)	37.68 (39.31–36.9)	.002
Area increase after fatigue (%)	53.5 (69.7–50.2)	35.2 (39.3–34.6)	.002
Stent outcomes N (%)			
PTFE changes	3	5 (100)	.114
PTFE breakpoints	–	3 (60)	.038
Stent fracture	–	2 (40)	.114
Stenosis	–	–	–
Technical failure	–	3	.038
Survival analysis			
Freedom from PTFE changes (M number of cycles)	40	5	.003
Freedom from stent breakpoint (M number of cycles)	50	25	.007

Abbreviations: ISLF, in situ laser fenestration; CMD, custom-made fenestrated endografts.

After fenestration creation, the short (2.33 mm) and long axis (4.03 mm) of the ISLF group were significantly (p=0.002) shorter compared to the custom-made fenestrations (5.80 and 5.83 mm, respectively). Both axes significantly (p=0.002) increased after bridging stent implantation and fatigue testing in the ISLF group, but remained lower compared to the CMD group.

The fenestration area of the CMD group was significantly larger than in the ISLF group before (24 mm^2^ vs 8.1 mm^2^; p=0.002) and after (37.68 mm^2^ vs 17.5 mm^2^; p=0.002). In both groups, the area increased after stenting and fatigue, in the ISLF group significantly more than in the CMD group (53.5% vs 35.2%; p=0.002).

**Figure 2.**
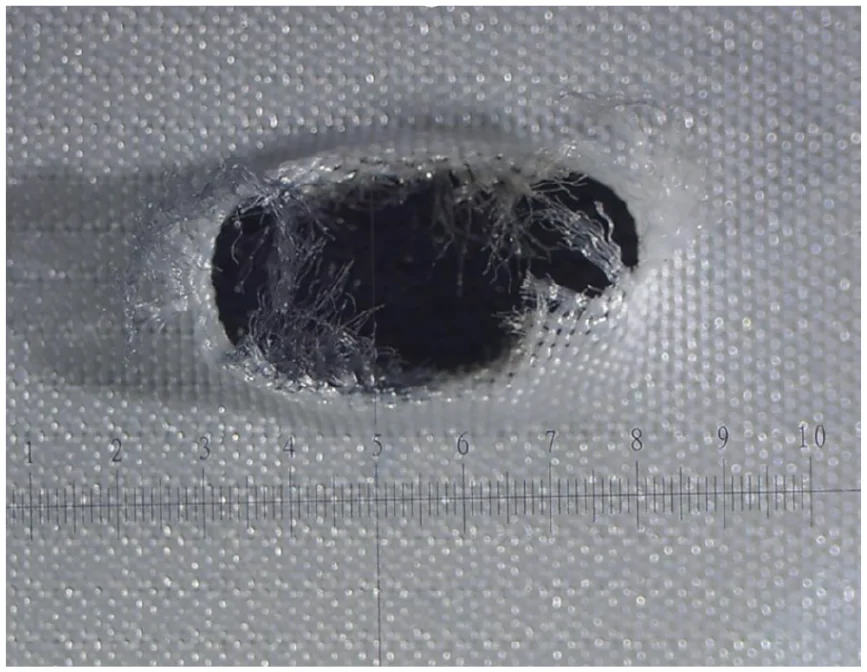
Fenestration of a RelayPro Dacron graft obtained with a 2.0 mm Turbo Elite OTW Laser Atherectomy catheter at a fluency of 45 mJ/mm^2^ and pulse rate of 25 Hz. Fraying and moderate tearing after ballooning with a 6 mm PTA catheter.

**Figure 3.**
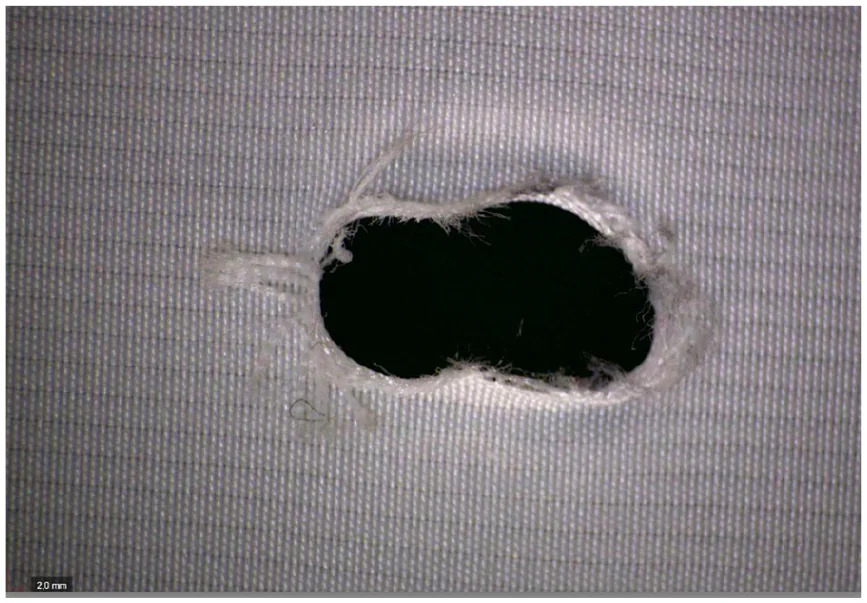
Same fenestration of a RelayPro Dacron graft after implantation of a 6 mm BeGraft and 50 million cycles, showing moderate fraying. The tearing observed after ballooning was not visible anymore.

Fraying was only found after ISLF creation, while the custom-made fenestrations did not show any damaged fabric fibers before and after fatigue. Before fatigue testing, tearing of the graft was observed in 3 of 5 fenestrations (Figure 2) of the ISLF group, but resolved after BSG implantation and did not become apparent during or after fatigue testing (Figure 3). No tearing was detected in the CMD group before and after fatigue testing.

Slight wear of the PTFE covering was found in all BeGraft-covered stents implanted in the CMD group and in 3 of 5 stents after ISLF. The freedom from initial PTFE changes (FIC) was statistically different between the 2 groups (p=0.003).

**Figure 4.**
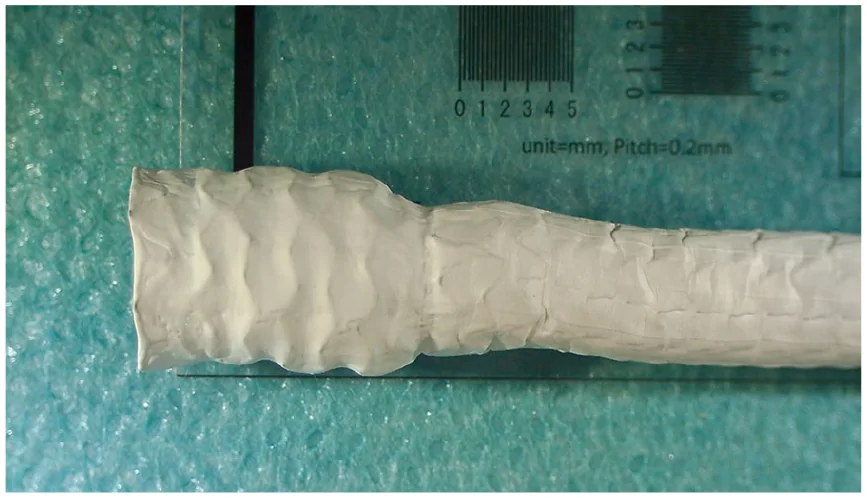
BeGraft stent removed from an in situ laser fenestration of a RelayPro Dacron graft after 50 million cycles without signs of PTFE damage.

**Figure 5.**
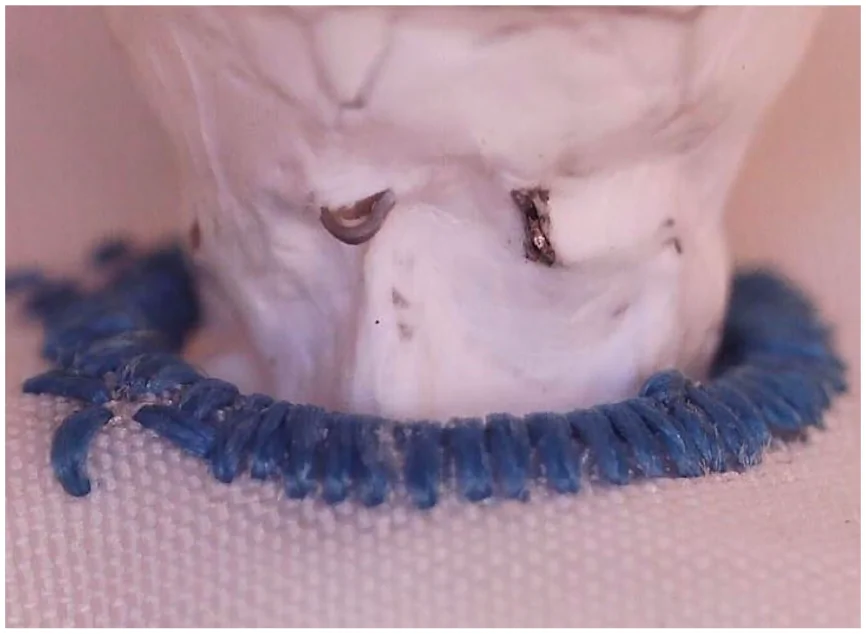
PTFE defects of a 6 mm BeGraft stent implanted in a custom-made fenestration after fatigue test using 40 million cycles.

No severe PTFE defects (breakpoint) were observed after fatigue testing in the ISLF group (Figure 4), while 3 of the 5 tested BeGrafts used for the CMD group revealed fabric defects with exposure to the stent metal (Figure 5). Accordingly, the FBP of the ISLF group was significantly higher compared to the CMD group (p<0.007).

**Figure 6.**
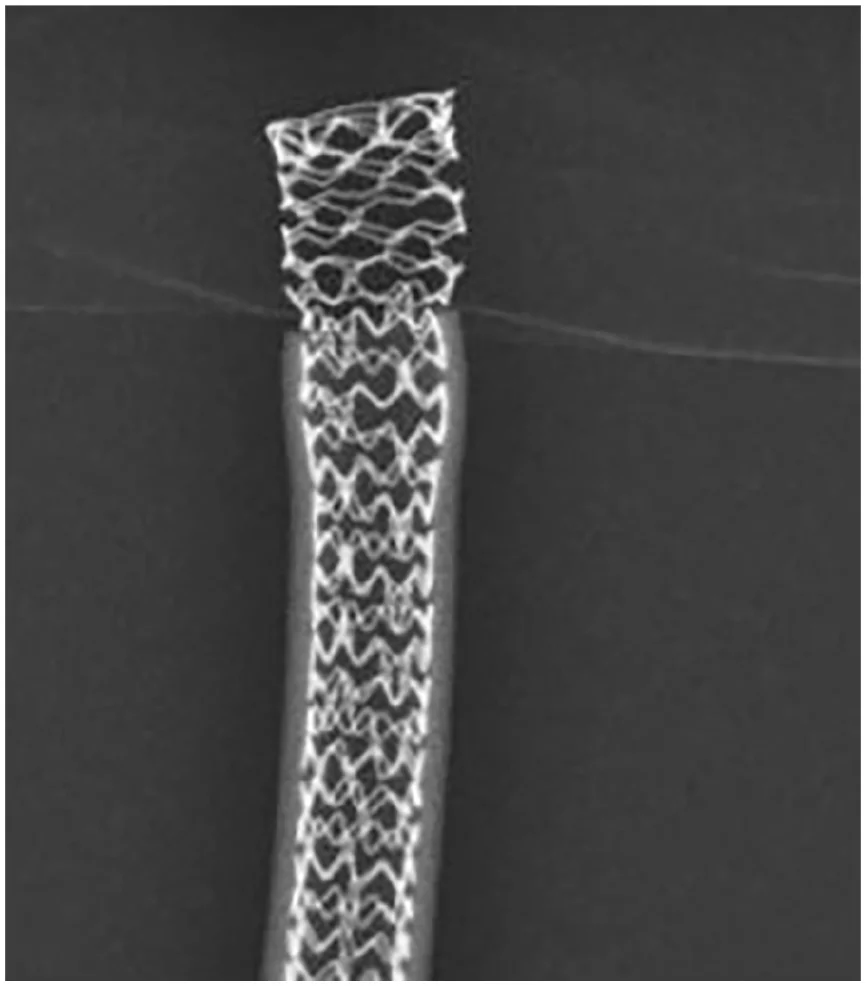
BeGraft stent explanted after in situ laser fenestration and fatigue up to 50 million cycles. The digital radiography shows the same structural changes of the endoskeleton observed after the flaring process without deterioration after 50 million cycles.

**Figure 7.**
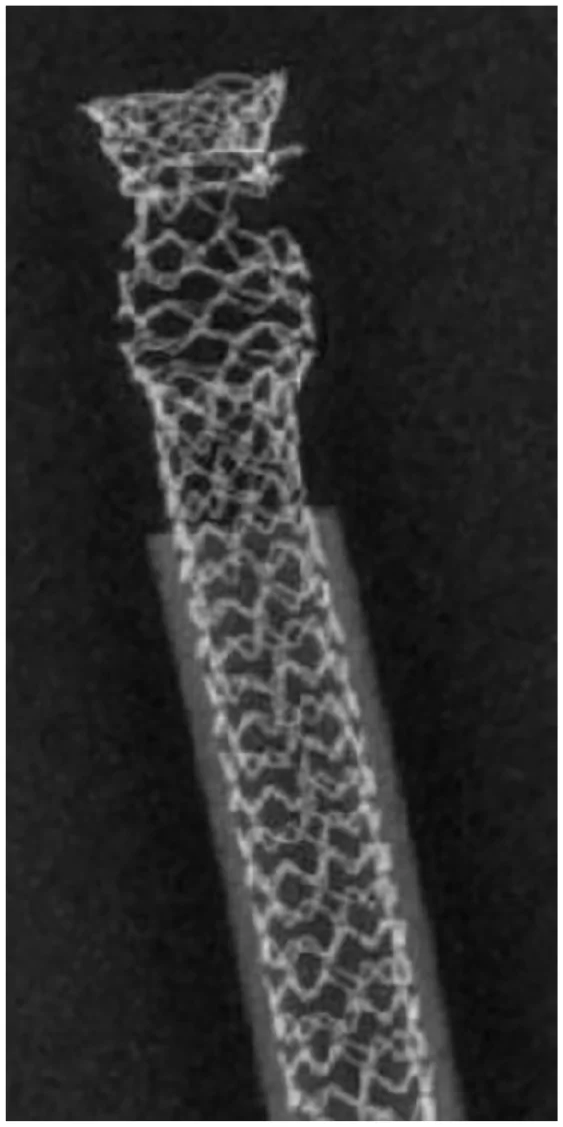
The digital radiography of a BeGraft stent used in a custom-made fenestration shows a partial fracture of the metallic frame after 40 million cycles.

**Figure 8.**
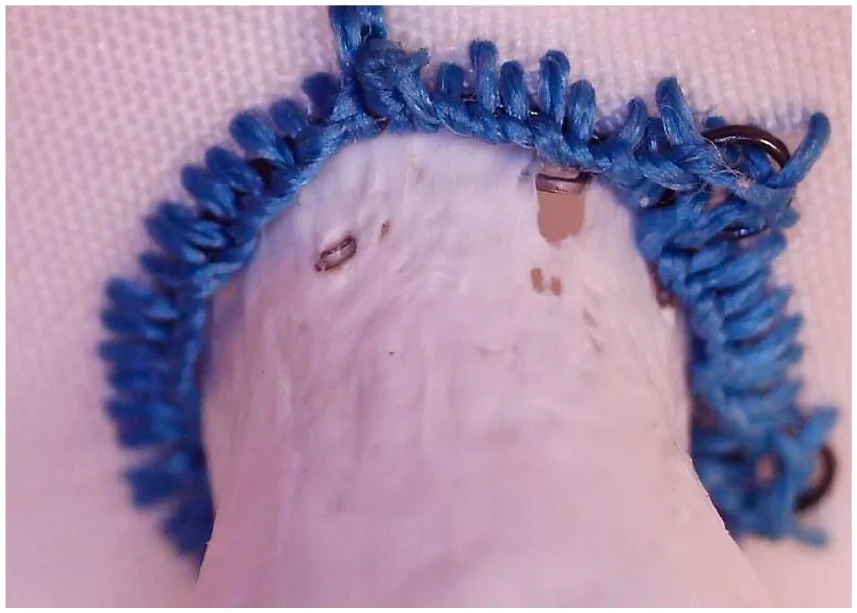
Severe damage to the PTFE layer of a BeGraft stent implanted in a custom-made fenestration after 40 million cycles. Small metallic component penetrates through the stent fabric.

No stent stenosis >30% was detected on digital radiography in both groups at the end of the fatigue test. All BeGraft stents implanted after ISLF showed structural changes of the endoskeleton directly after the flaring process without deterioration after 50 million cycles (Figure 6). Two of five BeGraft implants in the reinforced custom-made fenestrations presented partial fracture of the metallic frame at the level of the nitinol ring of the fenestration (Figure 7). Stent metal components penetrated the PTFE fabric (Figure 8).

With regards to the composite endpoint (stent fracture or stenosis, severe PTFE defects, or tearing after fatigue), the long-term technical failure was significantly higher in the fenestrated custom-made group. All results are summarized in Table 1.

## Discussion

This study evaluated the biomechanical performance of in situ laser-fenestrated endografts after fatigue. After 50 million cycles, the technical failure rate in the ISLF group was inferior to that observed with custom-made grafts. Notably, tearing was observed in 3 endografts of the ISLF group after ballooning. However, no tearing was visible after stenting and 50 million fatigue cycles and no stent fracture or stenosis was detected. This confirms the durability of ISLF, as reported by other authors, such as He,^6^ who evaluated the in situ laser technique in other endografts.

Immediately after manufacturing, the diameter and the area of the fenestrations obtained with the laser catheter were significantly smaller than those of custom-made fenestrations. Similarly, in other studies,^7^ reported reduced fenestration diameters in different endografts, ranging between 3.1 mm and 3.8 mm. The diameter could potentially be increased by using a cutting balloon instead of a conventional balloon. In our study, the fenestration size significantly increased after stenting and fatigue. Similar findings were reported by Jayet et al.,^8^ who observed an increase in fenestration surface area for all samples after 170,000 cycles. However, Eadie’s findings^9^ were more variable, showing size increases in the Talent fabric but not in the Endurant, Zenith, or ePTFE endografts. Lin^10^ noted that the Valiant endograft had the largest fenestration area, with the TX2 slightly larger than the Anaconda. In contrast, Ruthrauff et al^7^ found that in situ renal fenestrations did not expand beyond the target diameter despite cyclic fatigue.

Experimental in situ fenestrations often exhibit signs of fraying and tearing, as noted by several authors.^7,11,12^ This is more pronounced using multifilament weave endografts, such as Zenith, Anaconda, and Endurant. While fraying could be fixed after stent implantation, tearing may increase under fatigue and compromise endograft integrity. Similarly to other authors,^13^ we observed minimal tearing in three cases, which did not result in severe fabric damage after long-term fatigue. After 50 million cycles, the tears were fully incorporated into the fenestrations.

After stenting and fatigue, the shape of ISLF orifices became elliptical, aligning with findings from other authors.^6,9,12^ In one study,^6^ the lack of a circular fenestration shape did not appear to impact the stability of the stent within the fenestration.

To avoid potential damage to both the endograft and bridging stent, pullout tests were not performed. However, prior research has shown that the required strength to extract stents from ISLF fenestrations is high. Grima et al^14^ also demonstrated that BeGraft and BeGraft+ stents were firmly attached to fenestrations and provided a good seal in manual testing.

Slight PTFE changes were observed in the implanted BeGraft stents of both groups after fatigue. However, all-layer defects were identified only in the CMD group. It can be hypothesized that the absence of fenestration reinforcement may help preserve the integrity of the PTFE layer over time. Similar findings were reported by Torsello^5^, who observed all-layer defects in Advanta stents after fatigue, likely due to the penetration of the nitinol ring through the PTFE coverage.

Based on our findings, the risk of a Type 3B endoleak due to bridging stent damage may be more significant than a Type 3C endoleak caused by fenestration expansion. Transferring the results to CMD fenestration designs from other manufacturers is not possible due to the different concepts for creating the ring reinforcements, but it leaves room for further research.

Our study has several limitations. The number of evaluated fenestrations is limited by the high material costs of the stent used. However, as the study was not supported by any company, we can exclude any financial conflict of interest. In addition, the bench-top test can only partially replicate the in vivo conditions. The use of fabric sheets instead of tube grafts guarantees good comparability with previous and upcoming studies; however, there is a demand for more sophisticated tubular models. There is also a need for the examination of other BSGs currently available on the market.

The fatigue test used emulates the consequences of the respiratory-induced cyclic movements of the target vessels. Our model refers to renal arteries; mechanical stress on the proximal portion of the mesenteric arteries is expected to be lower. However, the biomechanical forces on bridging stents implanted in the human body are also affected by the blood pressure and morphologic changes of the aneurysm and of the endograft over time. Furthermore, such a fatigue test on ISLF should also be compared with other techniques (i.e. physician-modified endografts) as proposed by other authors.^15^

In conclusion, this study highlights the durability and biomechanical stability of ISLF endografts while identifying potential areas for improvement, such as fenestration shape optimization and alternative reinforcement strategies of the fenestration to mitigate long-term fabric damage.
